# An Outbreak of Pulmonary Tularemia in Slovenia in Summer 2024

**DOI:** 10.3390/epidemiologia6030051

**Published:** 2025-09-02

**Authors:** Irena Grmek Košnik, Kristina Orožen, Monika Ribnikar, Eva Grilc, Barbara Bitežnik, Miša Korva, Irena Zdovc, Jana Avberšek, Gorazd Vengušt, Maja Sočan

**Affiliations:** 1 National Institute of Public Health, 1000 Ljubljana, Slovenia; irena.grmek.kosnik@nlzoh.si (I.G.K.); kristina.orozen@nijz.si (K.O.); monika.ribnikar@nijz.si (M.R.); eva.grilc@nijz.si (E.G.); 2 National Laboratory for Health, Environment and Food, 4000 Kranj, Slovenia; 3 University Clinic Golnik, 4204 Golnik, Slovenia; barbara.biteznik@klinika-golnik.si; 4 Institute of Microbiology and Immunology, Faculty of Medicine, University of Ljubljana, 1000 Ljubljana, Slovenia; misa.korva@mf.uni-lj.si; 5 Veterinary Faculty, University of Ljubljana, 1000 Ljubljana, Slovenia; irena.zdovc@vf.uni-lj.si (I.Z.); jana.avbersek@vf.uni-lj.si (J.A.); gorazd.vengust@vf.uni-lj.si (G.V.)

**Keywords:** tularemia, pulmonary tularemia, outbreak, epidemiology

## Abstract

**Background**: Tularemia is a rarely identified disease in Slovenia. In summer 2024, we detected a tularemia outbreak in the Kranjsko-Sorško polje, located in North-Western part of Slovenia. **Aim:** To describe the epidemiological investigations and preventive measures to contain the outbreak. **Methods**: The patients with confirmed tularemia were interviewed. Serology and PCR was used for microbiological confirmation of tularemia and in some patients by isolation from blood or by RT-PCR. **Results**: The majority of confirmed tularemia cases in 2024 were infected in the geographically limited area in North-Western part of Slovenia (38/46). Tularemia was confirmed in two patients by isolation *Francisella tularensis* subsp. *holarctica* from blood or wound, in one by blood PCR, and in the others by serology. Most cases were associated with mowing or harvesting hay with intensive dusting. Twenty-eight (75.7%) out of 37 cases developed pulmonary tularemia. Sixteen cases were hospitalized. After confirming the outbreak, we alerted medical professionals in the region and the general public using the regional and national media and website of National Institute of Public Health. **Conclusions**: Endemic tularemia in Slovenia is associated with handling wild life and presents in ulceroglandular form. In the localized outbreak in year 2024 there was an extraordinary upsurge of pulmonary tularemia, with many of the cases initially investigated for lung cancer based on the radiology reports. Due to dry weather condition in summer 2024, excessive dusting associated with mowing the grass and handling hay resulted in inhalation of infective aerosols leading to the infection with *F. tularensis*.

## 1. Introduction

Tularemia is a zoonotic, re-emerging, potentially life-threatening infectious disease with diverse and often nonspecific clinical presentation, including fever, chills and malaise [1]. The disease occurs sporadically or in outbreaks. *Francisella tularensis*, the causative agent of tularemia, spreads over most of the northern hemisphere [1]. Taking a detailed history of exposure and a highly raised index of clinical suspicion are both necessary in order to take the appropriate diagnostic steps and therapeutic approach. In the environment, *F. tularensis* can survive for several months in soil, plants and water, which thus represent a source of infection for animals and humans. Some foods support long-term survival of *F. tularensis*, and it is resistant to the acidic environment in the stomach. Compared to other intestinal pathogens, *F. tularensis* is slightly more sensitive to high temperatures but survives freezing [1].

*F. tularensis* has four subspecies which differ in their pathogenicity [1,2]. *F. tularensis* subsp. *tularensis* (or Type A) is widespread mainly in North America and is the most virulent subspecies, with high mortality. The type A strains are further divided into subtypes A.I and A.II, which differ considerably in virulence, with the subtype A.I clade being the most virulent [2]. *F. tularensis* subsp. *holarctica* (or Type B) occurs in Europe, Asia, and North America. It causes a milder form of the disease, which is rarely fatal. *F. tularensis* subsp. *novicida* also causes a milder form of the disease. Finally, *F. tularensis* subsp. *mediasiatica* is mainly associated with infection in immunocompromised individuals [1].

The disease has been reported in many vertebrates (mammals, birds, amphibians, fish) and some invertebrates [3,4]. It was first isolated in 1911 as *Bacterium tularense* from rodents in the Tulare region of California. Despite its broad host range, tularemia is primarily a disease of animals of the orders *Lagomorpha* (rabbits) and *Rodentia* (rodents) [3,4]. Some data suggest that foxes and raccoons may also be important biological indicators for the presence of tularemia in wildlife populations [5]. In Central Europe, the brown hare is an important host, and occasionally the disease has also been detected in the mountain hare (*Lepus timudus*) [6].

The terrestrial and aquatic cycles of tularemia have been described in animals [7]. In the terrestrial cycle, rabbits and rodents play the most important role, while blood-sucking insects and arachnids play the role of indirect carriers (i.e., vectors). Infected animals excrete the bacteria with all their secretions, and thus contaminate the environment. The main route of infection has been found to be via the respiratory tract in brown hares and the gastrointestinal tract in mountain hares [4]. Cases of tularemia occur sporadically in the terrestrial cycle and occur after contact with a sick animal or after eating contaminated food [7]. Small rodents (e.g., voles) play an important role in the aquatic cycle, as they are susceptible to the disease but shed the bacteria into the environment over a long period of time due to the long duration of the disease [7]. The main source of infection is water, and via this route it can spread to Eurasian beavers (*Castor fiber*) and muskrats (*Ondatra zibethica*), which are also carriers and shedders of the bacteria [8]. Human infection via the aquatic cycle occurs in large outbreaks [7].

*F. tularensis* is transmitted to humans through a tick bite, contact with infected animals (hares, rabbits, voles, muskrats, beavers, etc.), and less frequently through the ingestion of contaminated food and water, and the inhalation of aerosols and dust, such as from agricultural work like mowing grass or loading hay. The infection occurs more frequently in certain professions, especially among hunters, foresters, farmers, veterinarians, butchers and in individuals who frequently spend active time in nature [6]. The main symptoms of the disease are fever, general malaise, headache, cough and enlarged lymph nodes. There may also be an ulcer on the skin, where the bacteria entered the body, or changes in the lungs. Pulmonary tularemia is the most severe form of tularemia, with high mortality. A recent systematic review of the worldwide literature on clinical features, antimicrobial treatment, and outcomes of human tularemia concluded that the ulceroglandular form of tularemia remains the most common (28.4%), followed by oropharyngeal (27.2%), glandular (16.0%), pneumonic (12.9%), oculoglandular (6.0%), typhoidal (5.1%), and meningitic (0.9%) disease [9]. In European studies, ulceroglandular tularemia accounted for the largest proportion of cases, while pulmonary tularemia was confirmed in approximately 10% of cases in recent years [10,11,12].

Tularemia is widely distributed throughout most of Europe and is endemic in Scandinavian countries. Surveillance of tularemia is mandatory in all reporting EU member states (MSs) and is mostly passive. In 2023, there were 1185 confirmed cases of human tularaemia, corresponding to EU notification rate of 0.27 cases per 100,000 population according to last published European Food Safety Authority (EFSA) and European Centre for Disease Prevention and Control (ECDC) joint report. In last five years (2019–2023) there were from 624 to 1321 tularemia cases reported in EU. In Europe, tularemia cases mainly occurred from July to November, but cases were observed all throughout the year. Monitoring data for *F. tularensis* in animals is voluntarily submitted to EFSA by EU MSs but only few countries provide the data. Human cases can signal the presence of *F. tularensis* in wild animals or the environment [13]. Animal surveillance is a valuable tool in understanding and managing tularemia, but it has several important limitations that affect its reliability, coverage, and usefulness for public health decision-making. Presence of *F. tularensis* in animals does not always correlate with human risk. Human exposure depends on many factors e.g., vector abundance (ticks, mosquitos, environmental conditions, human interaction with natural environment and wild or domestic animals [13]).

Tularemia is not a common disease in Slovenia, and the incidence rate varied between 0.0 and 2.6/100,000 inhabitants in the 10 years from 2014 to 2023. In total, only 102 cases were identified in this period, with 54 reported in 2021, when there was a waterborne outbreak in the Goriška region, caused by infected rodents contaminating small private water supply systems that were not properly maintained and monitored.

From 2014 to 2023, there were from zero to three cases reported annually in the Gorenjska region, followed by an unexpected increase in 2024. The relevant animal samples (e.g., hares) are very rarely examined in Slovenian microbiology laboratories, because it is difficult to obtain intact carcasses. To the best of our knowledge, *F. tularensis* has not yet been detected.

The aim of this paper was thus to describe the details of epidemiological investigations and actions to prevent new cases during this outbreak in 2024.

## 2. Materials and Methods

On 11 July 2024, the National Public Health Institute (NIPH) issued an alert about an increasing number of tularemia cases in the Gorenjska region in northern Slovenia. Reported cases were contacted and interviewed to obtain detailed epidemiological information using a structured epidemiological questionnaire. To classify the case, we used the EU case definition. A confirmed case was defined as a clinically compatible clinical presentation for ulceroglandular, oculoglandular, oropharyngeal, intestinal, pneumonic or thypoidal tularemia plus either isolation of *F. tularensis* from a clinical specimen or detection of *F. tularensis* nucleic acid in a clinical specimen or *F. tularensis* specific antibody response. Only confirmed cases were included in the analysis.

The standardized interview questionnaire for tularemia contains questions regarding demographic data, home address, date of onset of illness, hospitalization data and outcome of tularemia (recovered, died). The questionnaire includes classification of tularemia done by physician (ulceroglandular, oculoglandular, oropharyngeal, intestinal, pneumonic or thypoidal tularemia) and questions about exposure that could lead to the infection: contact with domestic or wild animals, tick bite, occupational exposure, type of water source used (municipal water system or own well), travel abroad during incubation period (to identify imported cases). Data on microbiological tests used to confirm tularemia are also part of standardized interview questionnaire.

Information about tularemia and recommendations for the medical community and general public were published on the NIPH’s website and communicated through local and national media. We used a One Health approach, i.e., an epidemiological investigation in cooperation with the Administration for Food Safety, Veterinary Sector and Plant Protection (AFSVSPP), Veterinary Faculty (VF), Institute of Microbiology and Immunology (IMI), Faculty of Medicine, University of Ljubljana and the University Clinic Golnik (UCG).

### 2.1. Microbiological Testing in Humans

The presence of specific IgG and IgM antibodies against *F. tularensis* was detected using an indirect immunofluorescence assay (*F. tularensis* MIF IgG and IgM kits; Fuller Laboratories, Fullerton, CA, USA) according to the manufacturer’s instructions. The IFA (Fuller Laboratories, Fullerton, CA, USA) detects antibodies directed against both lipopolysaccharide and outer-membrane protein antigens, and the slides also contain the *Brucella abortus* antigen as an internal control for detecting cross-reactivity. The cut-off point for both tests is 1:64. However, to confirm the diagnosis, we must detect either both IgG and IgM, or seroconversion, or a fourfold increase in antibody titres in the second sample.

For the purpose of molecular testing, DNA was extracted from patients’ EDTA blood, blood culture, lymph node aspirate samples and wound ulcer swabs using the EZ1&2 Virus Mini Kit v2.0 (QIAGEN, Hilden, Germany) following the manufacturer’s instructions. Subsequently, real-time PCR was performed targeting the ISFtu2 region of the *F. tularensis* genome using TaqMan Fast Virus 1-Step Master Mix (Applied Biosystems, Waltham, MA, USA) [14]. The nested PCR and sequencing of the *fopA* gene were performed on all the positive PCR samples to determine tularemia subspecies [15]. In addition, within the BSL-3 laboratory, we cultivated molecularly positive samples on chocolate agar and subsequently incubated then at 37 °C, pH 6.9 for a period of 2–4 days, as previously described [16,17].

### 2.2. Microbiological Testing in Veterinary Samples

The Faculty of Veterinary Medicine rarely tests sick and dead animals from the wild, because it is difficult for employees to access such animals [14]. Briefly, DNA was extracted from the internal organs by using an iHelix Complex kit (Institute of Metagenomics and Microbial Technologies, Ljubljana, Slovenia; https://www.ihelix.eu/ accessed on 17 August 2025) according to the manufacturer’s instructions. Real-time PCR assay was used for the amplification of the *ISFtu2* gene [9], using a TaqMan Universal Mastermix (Thermo Fisher Scientific, Waltham, MA, USA) and 7500 Fast Real-Time PCR System (Thermo Fisher Scientific, USA).

In 2024, two sick kittens, owned by a confirmed tularemia case, were tested at the IMI by serology and PCR in blood, as described above.

## 3. Results

### 3.1. Notified Tularemia Cases

**Figure 1:**
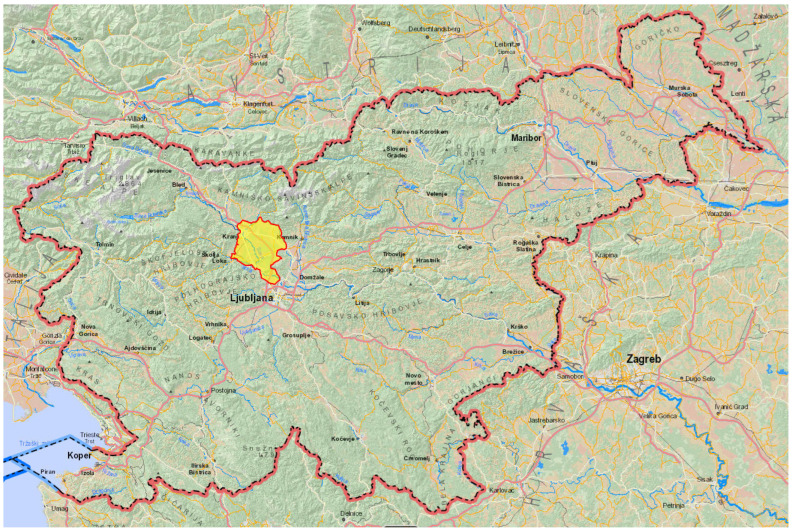
Geographic location of the pulmonary tularemia outbreak in Slovenia in 2025.

**Figure 2:**
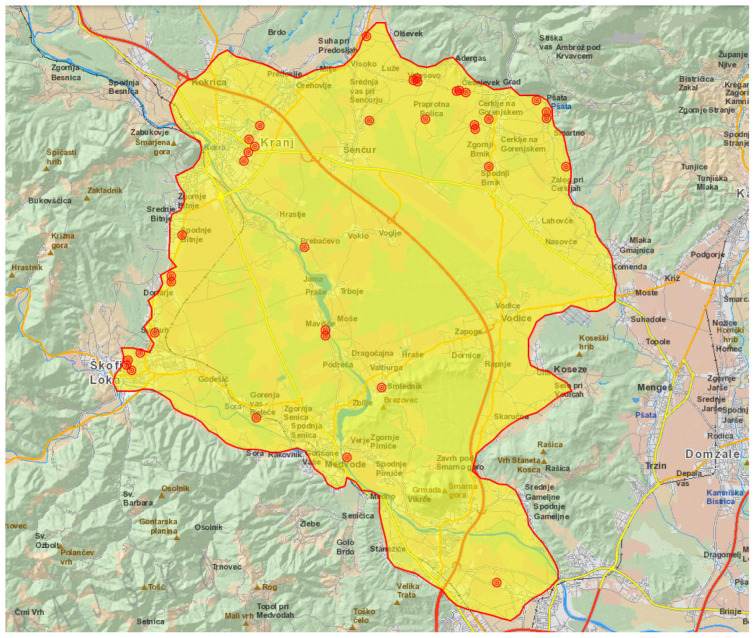
Distribution of tularemia cases in the Kranjsko-Sorško polje in 2025. One red dot represents one case.

Thirty-seven of the tularemia cases that were reported in 2024 were due to exposure in Kranjsko-Sorško polje in the north-western part of Slovenia (Figure 1 and Figure 2).

There were 29 male and eight female adult patients aged from 39 to 82 years, with an average age of 60.5 years. Nearly half of the cases (17) were pensioners, as expected according to the age structure, eight were farmers and seven were office workers. The remaining five patients were a construction worker, a carpenter, a truck driver, and a driving instructor, while one was unemployed (Table 1). As shown in Table 1, approximately two thirds were in contact with domestic animals, most often with cattle. Five patients reported a tick-bite during the incubation period, but as four of these developed the pulmonary form of the disease, it is more likely that the infection route was inhalation of *F. tularensis*. Most of the cases occurred in the summer months (June to August 29 cases, 78.4%). Two tularemia patients acquired the infection late in autumn, just before winter. One of them was cutting trees, peeling bark and making wood chips, the other was a farmer with multiple possible inhalation exposures.

Pulmonary tularemia was identified in 28 (75.7%) cases. Four patients had the ulcero-glandular form of tularemia, one patient the oculo-glandular form and one patient the glandular. Three patients suffered from prolonged febrile illness. Sixteen patients were hospitalized. There were no fatalities.

Mowing hay was the most frequently cited activity that most probably led to the infection with *F. tularensis*. Others linked to exposure were activities on a farm, gardening, walking in nature or having contact with domestic animals. Two patients could not recall any high-risk activities, although one of them often rode his motorbike during the time of cutting grass and harvesting hay in Kranjsko-Sorško polje, which might have led to inhalation of the bacteria and thus pulmonary tularemia.

In the patients with pulmonary tularemia malaise, fever and cough, dyspnea and chest pain dominated in clinical presentation. Chest computer tomography (CT) was performed with 14 patients and showed infiltrates with central necrosis and enlarged, centrally necrotic lymph nodes. Pleural effusion was present in three patients. Bronchoscopy was performed in six patients due to imaging suspicion of malignancy. Histology showed inflammation with necrosis. All patients with the pulmonary form of the disease were treated with ciprofloxacin. Regression of infiltrates on chest X-ray or chest CT was slow. In some patients there were still persistent changes after six months follow-up.

A total of 36 patients had diagnostic antibodies IgM and IgG against *F. tularensis* present in their first or second serum samples (Table 1). Using the real-time PCR, targeting the ISFtu2 region, led to the successful amplification of targeted DNA in one patient who at the time of testing was serologically negative. Subsequent sequence analysis revealed that all samples belonged to *F. tularensis* subsp. *holarctica*. Two serologically positive patients had *F. tularensis* isolated from blood and a wound on the neck, respectively. Three days after the inoculation on chocolate agar, small, light grey, reflective, soft, and easily emulsified colonies were observed, and *F. tularensis* was confirmed by real-time PCR.

### 3.2. Tularemia in Animals

During the epidemiological interview one patients stated that she had three kittens at home. All three had fallen ill and were examined by a veterinarian. One kitten died and was not available for testing. Blood was taken from two kittens and sent to the Laboratory for Diagnostics of Zoonoses and the WHO Laboratory, IMI. IgG antibodies against *F. tularensis* were present in the serum (1:320) of one of the two tested surviving kittens, while IgM antibodies were not detected. Real time PCR in blood samples was negative in both kittens.

In 2024, one hare was examined at the Veterinary Faculty and tested negative for *F. tularensis* by real-time PCR.

## 4. Discussion

The diagnosis of pulmonary tularemia can be challenging. Pulmonary tularemia may be an important radiological differential diagnosis to lung cancer. Many patients in the 2024 outbreak in Slovenia presented with several weeks of dry cough, weight loss and profuse night sweats. There was an obvious time lag between the first symptoms and microbiological confirmation of tularemia (Table 1). Chest CTs showed evidence of lymphadenopathy and consolidated lung masses with signs of necrosis. The radiological findings were described as suspicious of lung cancer. Serologies were positive for *F. tularensis* IgM and IgG, confirming the diagnosis of pulmonary tularemia. Serology is an easy way to confirm the diagnosis, in accordance with clinical or radiological suspicion of pulmonary tularemia [18,19].

Airborne transmission of *F. tularensis* with pulmonary manifestations of tularemia has been reported, but is rare compared to other clinical forms of *F. tularensis* infection [1]. Pulmonary tularemia typically results from inhalation of *F. tularensis*, but can occur as secondary pulmonary tularemia through hematogenous spread. Aerosolization of the bacteria is caused by some farming activities (e.g., moving the grass, brush cutting), wood chopping or hunting activities (e.g., skinning animal carcasses with contaminated fur) [6]. European studies have found pulmonary tularemia in only 3–12% of cases, and outbreaks are rarely described [20,21,22,23,24,25].

Dahlstrand et al. described an autumn-winter tularemia outbreak in northern Sweden in 1966, where the clinical data indicated that the infection was generally transmitted through inhalation of dust from hay contaminated with vole excreta [20]. At that time in Sweden, the clinical presentation of tularemia was mainly ulceroglandular or glandular, and occurred during summer. A more recent, retrospective observational study focusing on the northernmost county in Sweden revealed a high proportion (18%) of tularemia cases with respiratory symptoms (i.e., cough, dyspnea or pleuritic chest pain), with radiologically confirmed infiltrates or atypical findings on chest X-ray in half of the cases [11].

In the mid-1980s, Syrjälä et al. observed that in a tularemia epidemic in northern Finland, respiratory symptoms were present in 72% of the patients and nearly three quarters had abnormal chest films. Hilar adenopathy was the most common finding. Infection was airborne in most of the cases, and the patients acquired tularemia while mowing grass and harvesting hay. The authors concluded that airborne transmission may be an important source of infection during farming activities in endemic areas [26]. A Swedish case-control study comparing emerging and tularemia endemic areas revealed that the ulceroglandular form was more common in emergent areas, while pneumonic tularemia was more common in the disease-endemic area. Farming was a risk factor only in the disease-endemic area [22].

To identify risk factors for primary pneumonic tularemia, a case-control study of adults with this form of the disease was conducted after an outbreak in Martha’s Vineyard, USA, in 2000. In this, lawn mowing and brush cutting were identified as risk factors for primary pneumonic tularemia [27].

Siret et al. described an air-borne tularemia outbreak in 15 holiday-makers who spent their vacations in a converted mill in the Vendee region of France, which is an endemic area. The pulmonary form was present in 12 cases, suggesting the air-borne route of infection through aerosolized bacteria in contaminated dust or by contaminated particles present in dog fur [28]. Pulmonary infection was relatively mild compared to the more serious pneumonic infection caused by *F. tularensis* subsp. *tularensis* in North America [29].

In hunters, tularemia usually occurs after direct contact with infected animals and is clinically expressed in the ulceroglandular form. A tularemia outbreak with airborne transmission was reported in a group of hunters from Germany who were exposed during disembowelling and rinsing infected hares in water. They acquired the infection through inhalation of aerosolized particles, and in half of them pneumonia developed [30]. According to studies by Faber et al. and Appelt et al., tularemia is a rare but re-emerging zoonosis in Germany, and the pulmonary form was reported in only 11–12% of reported cases [25,31]. An upsurge of pulmonary tularemia was noticed in 2016 in northern Norway, with nearly half of all notified cases being the pulmonary form, a proportion considerably higher than in previous reports [18,19]. Wood chopping, farming, carpentry, hunting and other outdoor pursuits were all activities which lead to *F. tularensis* exposure [32,33]. The aerosolization of rodent excreta on woodpiles and on the wood of old houses was presumed to be the mode of transmission for those individuals who chopped wood or mended wooden houses [18,19].

*F. tularensis* has a broad spectrum of hosts and transmission routes, which makes it difficult to control. In the present study airborne transmission of *F. tularensis* seems likely but other routes of transmission are also possible, such as tick bites, which were mentioned by some patients and contact with domestic animals. Monitoring the presence of the bacterium in wildlife, insects and water sources provides important information that could be used as a basis for appropriate action if the incidence of the disease increases. Moreover, certain occupations—such as laboratory staff, hunters, farmers, veterinarians, and butchers—that come into frequent contact with reservoirs and vectors of the pathogen are at higher risk of the disease [34,35].

Preventing the infection during farming and outdoor activities can be challenging due to the presence of the bacillus in contaminated hay, soil and water, and preventive measures are directed towards minimizing the inhalation of contaminated aerosols. Mowing or using other machinery around animal carcasses, especially those of rabbits, muskrats, prairie dogs, and other rodents, should be avoided. Wearing a mask while using a mower or agricultural equipment may also help prevent breathing in any aerosolized particles, but more research is necessary to confirm this [36,37]. To prevent other forms of tularemia it is recommended to wear clothing that covers as much skin as possible while outdoors, especially in long grass or wooded areas. Wearing insect spray with DEET or picaridin is also strongly encouraged, as is checking oneself and one’s pets for ticks after being outside. Wearing gloves while handling animals—both living or dead—is also recommended. One should never pick up or touch a wild animal with one’s bare hands, and should always wash the hands thoroughly after handling any such animals. Meat should be cooked to safe temperatures, and the hands, surfaces and utensils should always be washed thoroughly after preparing food. It should noted that game meat can carry bacteria that cause tularemia, and drinking water should always be pretreated [36,37].

To contain any outbreaks of tularemia, we recommended the following:

- during mechanical farm work, the doors and windows of tractors should be closed at all times;
- farmers should protect themselves by wearing a surgical mask when handling hay indoors (especially if rodent excreta are present), while cleaning woodsheds and during other farm work where dust is intense;
- insect repellents should be used when engaging in outdoor activities.

Other general measures to reduce the risk of infection are also recommended:

- only safe drinking water from controlled water supply systems should be consumed;
- the access of rodents and insects to indoor spaces should be prevented;
- disinsection and deratization should be carried out regularly, especially if rodent excreta are present;
- raw meat and other foods of animal origin should be properly cooked before consumption;
- cross-contamination of foods (either with dirty hands, kitchen utensils, work surfaces or contaminated raw meat), and especially contamination of already cleaned and ready-made foods, should be avoided;
- direct contact with wild animals should be avoided, and touching wild animals—especially if they appear to be ill—is strongly advised against. Gloves should be used while handling dead wild animals. Animal carcasses should be sealed in a polyvinyl bag and properly disposed of, using gloves at all times. In the case of a large number of dead animals or the carcasses of larger wild animals, informing the veterinary hygiene service is warranted.

## 5. Conclusions

Until recently, tularemia was only rarely confirmed in Slovenia. The ulceroglandular form of the disease was most common, most often following a tick-bite or contact with wild life (and especially rabbits).

However, pulmonary manifestations of tularemia are possibly more frequent than previously reported. Our most notable finding was that the majority of patients were initially suspected of having lung cancer on CT, and thus pulmonary tularemia may be an important differential diagnosis to lung cancer in endemic areas. It is important to inform and alert both healthcare professionals and the general public about the possibility of pneumonia caused by *F. tularensis*, since it has not yet been included in the routine diagnostics for pneumonia.

## Figures and Tables

**Table 1:** Demographic, clinical, laboratory and epidemiological data of persons in the tularemia outbreak in Slovenia in 2024.

No.	Age/ Sex	Onset of Illness	Time Elapsed from Symptoms to Testing (in Days)	Exposure	Contact with Animals	Clinical Form	Hospitali-sation	IgM (second)	IgG (second)
1.	56/M	May	12	mowing hay	Yes	febrille illness	Yes	64	256
2.	53/M	May	14	mowing hay, farming	Yes	febrille illness	No	neg (>256)	256 (>256)
3.	57/M	May	73	nature walks	No	pulmonary	No	256	>1024
4.	63/F	May	39	contact with domestic animals	Yes	pulmonary	Yes	256	>256
5.	29/M	June	14	mowing hay, farming	Yes	pulmonary	Yes	>256	>256
6.	46/M	June	20	not identified	No	pulmonary	No	>1024	>1024
7.	56/M	June	16	mowing hay, farming	Yes	pulmonary	No	>256	>256
8.	59/M	June	12	mowing hay, farming	Yes	pulmonary	Yes	>256	>256
9.	52/M	June	33	not identified	No	pulmonary	No	>1024	>1024
10.	67/M	June	35	mowing hay	Yes	pulmonary	No	1024	>1024
11.	71/M	June	30	mowing hay, gardening	No	pulmonary	No	1024	>1024
12.	74/M	June	59	gardening	Yes	ulceroglan	Yes	1024 *	>1024
13.	82/M	June	37	mowing hay, gardening	No	pulmonary	Yes	>1024	>1024
14.	62/M	June	85	mowing hay, farming	Yes	pulmonary	No	256	>256
15.	59/M	June	97	farming	Yes	pulmonary	No	256	>1024
16.	66/M	July	13	beekeeping	Yes	pulmonary	Yes	1024	256
17.	62/M	July	16	gardening	Yes	pulmonary	No	1024	256
18.	67/M	July	18	gardening	No	pulmonary	Yes	>1024	>1024
19.	62/F	July	24	mowing hay	Yes	pulmonary	No	512	>1024
20.	43/F	July	4 (HT)	gardening	No	pulmonary	Yes	>256 **	>256
21.	68/F	July	21	nature walks	No	pulmonary	No	>1024	>1024
22.	77/M	July	33	mowing hay, gardening	Yes	pulmonary	Yes	>1024	>1024
23.	77/M	July	7 (PCR)	mowing hay, farming	Yes	pulmonary	No	neg #	neg
24.	80/F	July	35	nature walks	Yes	ulceroglan	Yes	neg. (neg.)	2048 (2048)
25.	39/M	July	29	mowing hay, farming	Yes	pulmonary	Yes	>1024	>1024
26.	42/M	July	12	mowing hay	Yes	pulmonary	Yes	neg (>256)	64 (>256)
27.	64/M	July	11	mowing hay	No	pulmonary	No	>256	>256
28.	43/F	August	10	contact with domestic animals	Yes	glandular	No	neg (>256)	1024 (>256)
29.	43/M	August	13	farming, collecting straw	Yes	oculoglan	No	neg. (neg.)	256 (8192)
30.	76/M	August	21	gardening, grafting trees	No	pulmonary	Yes	128	>256
31.	69/F	August	39	gardening	No	ulceroglan	No	>256	>256
32.	78/M	August	21	farming	Yes	febrille illness	No	128	>256
33.	69/M	August	0 (neg)	gardening	No	pulmonary	No	neg (1024)	neg (>1024)
34.	60/M	September	16	farming	Yes	pulmonary	No	neg (>1024)	>256 (>1024)
35.	57/F	September	24	gardening	No	ulceroglan	Yes	>256	>256
36.	59/M	December	33	farming	Yes	pulmonary	No	1024	>1024
37.	52/M	December	116	timber work	No	pulmonary	Yes	1024	>1024

* isolation from wound, ** isolation from haemoculture, # PCR positive in blood sample, ulceroglan = ulceroglandular form, oculoglan =oculoglandular form.

## Data Availability

Data supporting reported results are available at researches I.G.K. and K.O.
